# Heterophonic speech recognition using composite phones

**DOI:** 10.1186/s40064-016-3332-9

**Published:** 2016-11-24

**Authors:** Ashraf Alkhairy, Afshan Jafri

**Affiliations:** 1 King Abdul Aziz City for Science and Technology, Riyadh, Saudi Arabia; 2 King Saud University, Riyadh, Saudi Arabia

**Keywords:** Syllables, Phonemes, Heterophones, Speech Recognition, Arabic

## Abstract

Heterophones pose challenges during training of automatic speech recognition (ASR) systems because they involve ambiguity in the pronunciation of an orthographic representation of a word. Heterophones are words that have the same spelling but different pronunciations. This paper addresses the problem of heterophonic languages by developing the concept of a Composite Phoneme (CP) as a basic pronunciation unit for speech recognition. A CP is a set of alternative sequences of phonemes. CP’s are developed specifically in the context of Arabic by defining phonetic units that are consonant centric and absorb phonemically contrastive short vowels and gemination, not represented in the Arabic Modern Orthography (MO). CPs alleviate the need to diacritize MO into Classical Orthography (CO), to represent short vowels and stress, before generating pronunciation in terms of Simple Phonemes (SP). We develop algorithms to generate CP pronunciation from MO, and SP pronunciation from CO to map a word into a single pronunciation. We investigate the performance of CP, SP, UG (Undiacritized Grapheme), and DG (Diacritized Grapheme) ASRs. The experimental results suggest that UG and DG are inferior to SP and CP. For the A-SpeechDB corpus with MO vocabulary of 8000, the WER for bigram and context dependent phone are: 11.78, 12.64, and 13.59 % for CP, SP_M (SP from manual diacritized CO), and SP_A (SP from automated diacritized MO) respectively. For vocabulary of 24,000 MO words, the corresponding WER’s are 13.69, 15.08, and 16.86 %. For uniform statistical model, SP has a lower WER than CP. For context independent phone (CI), CP has lower WER than SP.

## Background

A standard automatic speech recognition (ASR) system consists of a language model (LM) that governs the sequence of words in an utterance, a dictionary that maps words into sequences of pronunciation units, and Hidden Markov Models (HMMs) corresponding to pronunciation units that stochastically model acoustic events (Huang and Acero 2001).

The pronunciation units could correspond to either a phoneme (an individual phonetic segment) or to a syllable (a structured sequence of phonemes), with the choice depending on characteristics of a given language’s phonological system. In an ASR system that uses the phoneme, phonetic segments could correspond to O(N) context independent Monophones for a language with N phonemes, or be refined to correspond to as many as O(N^3^) context dependent Triphones. In a syllable-based ASR system, the pronunciation units would correspond to a language’s inventory of syllables.

Training of HMMs is conducted on phonetic transcriptions of speech utterances, which are derived from orthographic transcriptions using ortho-phonetic mapping. One important problem that arises during the training phase is the ambiguity posed by heterophones—words that have the same orthographic representation but different pronunciations (e.g., in English, the noun “bow”, referring to a weapon, and the verb “bow”, referring to a gesture of respect) (Wikipedia 2016).

This problem is typically addressed using the alignment method (Soltau et al. 2007), which initially estimates HMMs using arbitrary single pronunciations of words. This method then iteratively aligns the pronunciation choices with the estimates of HMMs, to yield better single pronunciations of words in the utterance and more accurate HMMs.

Languages with a significant number of heterophones, however, require solutions to the challenges faced during HMM training that go beyond the alignment method (Demeechai and Makelainen 2001). Among these languages are Hebrew, in which the written form of a word typically has three pronunciations, and Arabic, in which the written form of a word has on average 5–10 pronunciations.

Hebrew and Arabic (as well as other languages) have “deep” orthographies and omit short vowels that are frequently interspersed between consonants, as well as phonemically contrastive consonant gemination. For languages with deep orthographies, generic grapheme-phoneme mappings cannot determine the pronunciation of words without additional contextual rules.

As an illustration, the three English words ‘bin’, ‘ban’, and ‘bun’ would all be written as ‘bn’. The absence of certain types of phonemes in the orthographic representations of words in these languages results in large numbers of heterophones, and thus we refer to these languages as “heterophonic”. Here, the focus will be on Arabic, although the proposed solutions are intended to apply more generally to heterophonic languages.

Current literature solves the heterophone HMM training problem using three broad approaches:

1. Diacritize the words and then model them phonemically (Kirchhoff and Vergyri 2005). We refer to this approach as the Simple Phoneme (SP) method, since the standard phoneme is the pronunciation unit. This approach pre-processes the orthographic transcriptions of training utterances by adding diacritics to words so that a diacritized word has a single pronunciation. It then computes the HMMs of the Monophones (or Triphones). The SP category may be divided into manual diacritization (Abushariaha et al. 2012), and automatic diacritization (El-Desoky et al. 2009; Mangu et al. 2011). We denote these methods as Manual Diacritization (SP_M) and Automated Diacritization (SP_A). A hybrid approach bootstraps diactritized models with a small manually diacritized subset (Lamel et al. 2009). Training based on automatic diacritization is not robust because the spoken word may differ from the intended pronunciation, or it may deviate from the correctly derived syntactic and semantic version due to a lack of diacritics during prompts for utterance recording (Habash and Roth 2009). Training based on manual diacritization is quite labor intensive.
2. Retain the undiacritized words and handle multiple pronunciations during training. The second approach modifies the standard training approach (implemented in many training tools, including HTK) to handle multiple pronunciations (Soltau et al. 2009). It retains an undiacritized transcription of training utterances and creates a pronunciation dictionary of undiacritized words along with all possible pronunciation variants.
3. Model the words graphemically. This approach has graphemes as pronunciation units (Billa et al. 2002; Anumanchipalli et al. 2006; Magimai-Doss et al. 2003a, b), and thus computes HMMs for graphemes, rather than SPs. We refer to this method as the Undiacritized Grapheme (UG) method because the pronunciation units are specifically Undiacritized Graphemes. The grapheme-based HMMs would in general not meet the objectives of phonetically aligned HMMs. For languages with a deep orthography, the Grapheme approach can be inferior to the SP (Simple Phoneme) approach by up to 10 % word error rate (WER), depending on task and complexity of mapping from orthography to pronunciation (Kanthak and Ney 2002; Magimai-Doss et al. 2003a, b). This difference arises because the SP approach incorporates language-specific knowledge. The SP approach would thus be expected to have superior performance compared to the UG method for Arabic, a language with a deep orthography and moderately complex orthography-pronunciation mapping.

To address the problem of HMM training for heterophonic languages (i.e., those with deep orthographies that produce large numbers of heterophones), this paper develops the concept of a Composite Phoneme (CP)—a set of alternative sequences of phonemes, such as a syllable with multiple vowels choices. A word would then typically result in single pronunciation in terms of a CP sequence. Using CPs as pronunciation units, this approach defines O(N) Composite Phonemes for a language with N phonemes, with the objective of yielding a small number of compact and disjoint HMMs that are aligned with acoustic features. We refer to this as the Composite Phoneme (CP) method.

We design CP to be a phonetic pronunciation unit with suitable amount of ambiguity for purposes of ASR in languages like Arabic and Hebrew. In order to accomplish this, disambiguating and ambiguating factors are discerned, disambiguating features retained as contrastive factors in the pronunciation unit, and other factors transferred as ambiguity to the pronunciation unit. Note that both pronunciation unit and ambiguity are defined within the context of objective and language.

If the objective is to extract meaning from sounds, then SP is the appropriate pronunciation unit because it is the smallest contrastive unit that changes meaning—while incorporating ambiguity arising from irrelevant features such as pitch. If the objective is natural speech synthesis, then SP is not suitable because a pronunciation unit must disambiguate between the same SP with distinct intensity, duration, pitch, and glottal characteristics. If the objective is ASR, then the pronunciation unit must disambiguate factors relevant to the orthographic representation of sounds while retaining ambiguity in irrelevant factors, such as prosody.

As our goal is to design a phonetic pronunciation unit for a language like Arabic with an objective of building an ASR, short vowels and gemination are ambiguating factors because recognition performance for Arabic uses undiacritized orthography. If recognition performance used diacritic orthography (which is not the case), then short vowels and gemination would be disambiguating factors, and hence CP would not be an appropriate pronunciation unit.

Ambiguity also exists in the definition of SP. An example is SP for English which includes ambiguity concerning velarization, gemination, stress, lengthening, and aspiration, whereas SP for Arabic, Hindi, and Mandarin disambiguate phonemes based on these factors, and hence are factors in defining their simple phonemes.

A Simple Phone ASR must discern ambiguous and disambiguous factors for the intended language. We would want to design ambiguity into the definition of a pronunciation unit. There is no point in building an English ASR that contrasts between short and long vowels, velarized and non-velarized sounds, and singleton and geminate consonants. Also, there is no need to incorporate the ability to contrast between a low pitch or creaky voice and a high pitch and modal voice for ASR.

Intuitively, CP is a pronunciation unit for a listener who cannot differentiate between a singleton consonant and its geminate version and among short vowels. It may also be thought of as a pronunciation unit for a speaker who is lax in pronouncing singleton/geminate versions of a consonant and various versions of short vowels.

Our objective is to design and run experiments to compare performance of CP with SP_M, SP_A, DG, UG. We utilize approaches such as Gaussian mixture HMM model, maximum likelihood estimation of HMM model and triphone state-tying, and conduct experiments with commonly used HTK values provided in Table 1 across all these pronunciation units.

**Table 1:** HTK parameter values used in experiments

Item	Value
Signal Processing	Frame period: 10 ms, Hamming window size: 25 ms, first order pre-emphasis coefficient: 0.97
Feature vector	Cepstral lifting coefficient: 22, filterbank channels: 26, 12 MFCC coefficients and 1 energy + delta + acceleration for total of 39 coefficients
HMM topology	5 state non-skip left-to-right with diagonal covariance
HMM Variance	Floor on variance estimated: 0.01 * global covariance
HMM training and realignment	Start with pruning beam width at 250 and increment at 150 for a maximum of 1000
Triphone Cluster	Minimum number of frames allocated to any cluster: 100
Decision tree splitting	Split cluster into two until increase in log likelihood falls below 350
Data driven clustering	Greatest distance between any two states in cluster: 100
Decoding	Word insertion log probability: 0.0; Language model grammar scale factor: 1.0

A state-of-the art system fine tunes parameters values for a specific pronunciation unit to optimize its performance, followed by refinements that include adaptive training, lattice based discriminative training, and deep neural networks. Methods include maximum likelihood linear regression (MLLR) to linearly transform model parameters for speaker adaptation (Leggetter and Woodland 1995; Povey and Saon 2006), maximum mutual information (MMI) to compensate for deviation from HMM model, minimization of phone error (MPE) to reduce Bayes’ risk (Povey and Woodland 2002; Kuo et al. 2011) and deep neural networks to model complexity (Hinton et al. 2012; Dahl et al. 2012). Recent state-of-the-art Arabic ASRs for SP_A conduct fine tuning and refinement resulting in significant improvement in WER (Cardinal et al. 2014).

## Framework

A summary of Arabic phonemes and their corresponding orthographic representations illustrates the issue of heterophonic languages (Alghamdi et al. 1997). Table 2 lists the 46 Arabic characters and their Roman transliterations using the Buckwalter scheme. Table 2 also lists the generic mapping from characters to phonemes. Arabic consists of three short vowels, their long versions, 28 singleton consonants, and their geminate versions—for a total of 62 phonemically contrastive sounds. The last columns also present generic mapping from characters to Composite Phonemes (CPs) (to be explained later).

**Table 2:** Generic Arabic character-phoneme mappings

Ch	Rm	Phone	CP	Ch	Rm	Phone	CP
ــَـ	a	a		ـْـ	o	ϕ	
ـُـ	u	u		ـّـ	~	Geminate	
ـِـ	i	i		ـ’ـ	G	a:	{a:}
ـًـ	F	a 0	{0}	ذ	*	?	{?}
ـٌ	N	u 0	{0}	ر	r	6	{6}
ـٍـ	K	i 0	{0}	ز	z	A	{A}
ء	‘	-	{-}	س	s	@	{@}
أ	>	-	{-}	ش	$	B	{B}
ؤ	&	-	{-}	ص	S	@^√^	{@^√^}
إ	<	-	{-}	ض	D	$^√^	{$^√^}
ئ	}	-	{-}	ط	T	#^√^	{#^√^}
ا	A	a: or ϕ or -	{a:} or ϕ or {-}	ظ	Z	?^√^	{?^√^}
ى	Y	a:	{a:}	ع	E	M	{M}
آ	|	- a:	{-} {a:}	غ	g	K	{K}
ب	b	"	{"}	ف	f	<	{<}
ة	p	# or N	{#} or {N}	ق	q	+	{+}
ت	t	#	{#}	ك	k	)	{)}
ث	v	>	{>}	م	m	.	{.}
ج	j	C	{C}	ن	n	0	{0}
ح	H	L	{L}	ه	h	N	{N}
خ	x	J	{J}	و	w	f or u:	{f} or {u:}
ل	l	W or ϕ	{W} or ϕ	ي	y	U or i:	{U} or {i:}
د	d	$	{$}				

Phonemes are represented by IPA symbols. CPs are represented by curl bracketed IPA symbols. Bold IPA symbols in text denote gemination (not shown in table)

Buckwalter transliteration (Roman) is used, with extra symbol added for Madd

Note that a character may be mapped into a single phoneme, multiple phonemes, or a sequence of phonemes, and more than one character may map onto the same phoneme. Thus, in contrast to “phonemic” languages, in which shallow orthographies produce largely one-to-one grapheme-to-phoneme mappings, the generic mappings in Arabic cannot determine the pronunciation of a word without additional contextual rules.

Arabic is generally written in Modern Orthography (MO), which excludes the six diacritic characters in the top three rows of Table 2. Those diacritics are related to three short vowels ‘a’, ‘u’, ‘i’ (), consonant gemination ‘~’ ( ـّـ ), and a long vowel ‘G’ ( ـ'ـ ). The three diacritic Tanween characters ‘F’, ‘N’, and ‘K’ () may only occur at the end of a word; this is also true of ‘p’ (ة), which may also be followed by a diacritic. Classic Orthography (CO), which is used mainly in classical texts, includes those six diacritics. Consequently, a word written in MO corresponds to a number of words written in CO. The term diacritization of a word refers to conversion of MO to CO by addition of the six diacritic characters. The terms CO and MO are used rather than diacritized and undiacritized text because MO does contain Tanween, and because these terms also avoid confusion with the terms Undiacritized Grapheme (UG) and counterpart Diacritized Grapheme (DG) pronunciation units/methods used in the paper.

An example in Arabic is the word ‘علم’, transliterated in MO as ‘Elm’. Some of the alternatives of the example word ‘Elm’ علم are given below, illustrating the CO, Roman transliteration, phonemic pronunciation and meaning (Alghamdi et al. 2005): عِلْمْ ‘Eilmo’/Milm/(science), عَلـَمْ ‘Ealamo’/Malam/(flag), عـَلـَّمَ ‘Eal~ama’/Ma**l**ama/(he taught), عُـلِـمَ ‘Eulima’/Mulima/(known), عُـلـِّمَ ‘Eul~ima’/Mu**l**ima/(has been taught), عـَلـِمَ ‘Ealima’/Malima/(he knew). As expected, a single representation in MO corresponds to these several unambiguous representations in CO.

## The composite phoneme

The proposed Composite Phoneme (CP) concept allows for training of HMMs corresponding to O(N) Phonetic units that cater to the ideal objectives of the HMMs modeling pronunciation units to be compact, disjoint, stable, small in number, and aligned to phonetic units (Jelinek 1997).

This concept does not require alteration to the standard training methodology. It does not involve diacritization of the training transcription in MO, making it robust against unreliable and inconsistent diacritization, and does not need a dictionary in CO, making it feasible. Furthermore, it does not require a large number of phonetic units, which allows it to be trained on a reasonable size corpus of training data.

To show how CP structure makes use of language-specific knowledge to more closely approximate phonetic models, Arabic serves as the case study in this paper. It’s important to note that a CP in Arabic is not an arbitrary unit of analysis. Rather, as shown below, it’s closely connected to the structure of syllables, reflects the interface between the phonology and orthography of the language, and utilizes the strong consonantal nature of Arabic.

An important characteristic of Arabic is that syllables start with a consonant. For example, in العرب (AlErb), the character ‘A’ is pronounced as the sequence of a glottal stop consonant (-) and a vowel, rather than a vowel alone. In general, a consonant may be geminated and followed by any of the vowels.

A CP is constructed by clustering a singleton consonant (C), its geminate counterpart (CC), and all the consonant short vowel sequences, for both the singleton and geminate versions of the consonant (CV, CCV), into a single phonetic unit. In other words, a Composite Phoneme in Arabic can encompass these four basic *structural* units: {C, CC, CV, CCV}, with the actual number of units comprising a CP determined by the number of short vowels that can be linked to a given consonant.

Our objective is to define O(N) pronunciation units that contribute towards attaining single pronunciations for words in MO and the desired characteristics of HMMs. In an ASR system, an MO word is represented by a sequence of CPs. We develop CPs incrementally in the following paragraphs. The resulting CP definitions in terms of phonemes are provided in Table 3.

**Table 3:** Composite phoneme definitions

CP	Definition in terms of phonemes	CP	Definition in terms of phonemes
a:	a:	6	6 | 6 a | 6 u | 6 i | 6 | 6 a | 6u | 6 i
u:	u:	A	A | A a | A u | A i | A | A a | A u | A i
i:	i:	@	@ | @ a | @ u | @ i | @ | @ a | @ u | @ i
f	f | f a | f u | f i |f | f a | f u | f i	B	B | B a | B u | B i | B | B a | B u | B i
U	U | U a | U u | U i | U | U a | U u | U i	@^√^	@^√^ | @^√^ a | @^√^ u | @^√^ i | @^√^ | @^√^ a | @^√^ u | @^√^ i
0	0 | 0 a | 0 u | 0 i | 0 | 0 a | 0 u | 0 i	$^√^	$^√^ | $^√^ a | $^√^ u | $^√^ i | $^√^ | $^√^ a | $^√^ u | $^√^ i
-	- | - a | - u | - i | - | - a | - u | -i	#^√^	#^√^ | #^√^ a | #^√^ u | #^√^ i | #^√^| #^√^ a | #^√^u | #^√^ i
"	" | " a | " u | " i | " | " a | " u | " i	?^√^	?^√^ | ?^√^ a | ?^√^ u | ?^√^ i | ?^√^ | ?^√^ a | ?^√^u | ?^√^ i
#	# | # a | # u | # i | # | # a | # u | # i	M	M | M a | M u | M i | M | M a | M u | M i
>	> | > a | > u | > i | > | > a | > u | > i	K	K | K a | K u | K i | K | K a | K u | K i
C	C | C a | C u | C i | C | C a | C u | C i	<	< | < a | < u | < i | < | < a | < u | < i
L	L | L a | L u | L i | L | L a | L u | L i	+	+ | + a | + u | + i | + | + a | + u | + i
J	J | J a | J u | J i | J | J a | J u | J i	)	) |) a |) u |) i |**)** |**)** a |**)** u |**)** i
W	W | W a | W u | W i | W | W a | Wu | W i	.	. | . a | . u | . i | **.**| **.** a | **.** u | **.** i
$	$| $ a | $ u | $ i | $ | $a | $ u | $i	N	N | N a | N u | N i | **N** | **N** a | **N** u | **N** i
?	?| ? a | ? u | ? i | ? |? a | ? u | ? i		

The International Phonetic Alphabet (IPA) symbol denotes a phoneme, a bold IPA symbol denotes gemination, and a IPA symbol (with curl bracket omitted) notates a CP

The steps for constructing CPs are as follows:

1. Define the CPs {a:} = /a:/, {u:} = /u:/, and {i:} = /i:/ as the phonemes themselves, because each of the long vowels is represented orthographically.
2. Define CPs for the approximants {f} = /f/ and {U} = /U/. Although these consonants are represented orthographically in the same way as the vowels /u:/ and /i:/, they are acoustically distinct, and hence are defined separately.
3. Consider the case of a singleton consonant phoneme and its geminate counterpart. Although the distinction is phonemic (i.e., gemination of a consonant can be the sole distinction between two words in Arabic), it is not represented orthographically; we therefore assign a single CP for both the singleton consonant and its corresponding geminate. As an example, {0} = /0/ | /**0**/ represents *both* the singleton consonant and its geminate counterpart. Although the resulting HMM has more variability, it remains coherent because the singleton and geminate forms of a phoneme are acoustically similar.
4. Consider the phoneme sequence /0 a/. As short vowels are unrepresented in MO, a potential phonetic unit is {0_a_} = /0a/. The HMM for this phonetic unit meets our objectives because CV is a syllable in Arabic (it is the most frequent syllable),where C is a consonant and V is a short vowel. We expect the HMM for {0_a_} to be only slightly longer than that for /0/ because the succeeding vowel is short in duration (Olive et al. 1993). We do not define phonetic units of the form /a 0/, /u 0/, or /i 0/ because syllables in Arabic do not start with a vowel.
5. Consider the phonetic units {0_a_} = /0 a/, {0_i_} = /0 i/, {0_u_} = /0u/, with the consonant followed by the short vowels. As these phonetic units are not contrastive orthographically, a potential phonetic unit is {0} = /0a/ | /0i/ | /0u/. Most of the states of the HMM of {0} are expected to be coherent because they correspond to the same consonant, with the last state(s) having some variability due to the short vowels.
6. Consider the addition of /0/ to {0}, making it {0} = /0/ | /0 a/ | /0 i/ | /0 u/. The HMM of the original {0} = /0 a/ | /0 i/ | /0 u/ can be modified to incorporate /0/ because the state(s) corresponding to the short vowel are expected to have a reduced impact on the definition of the HMM.
7. Combine the consonant-centric phonetic units with their consonant geminate counterparts and define the CP {0} = /0/ | /0 a/ | /0 i/ | /0 u/ | /**0**/ | /**0** a/ | /**0** i/ | /**0** u/. Thus the CP incorporates both the singleton and geminate counterpart of a consonant, and the absence or presence of short vowels. This definition is generalized to other consonants, as shown in Table 3.

The definitions of the CPs in terms of phonemes in Table 3 are used to determine the generic mappings between the characters and CPs in Table 2. A word may have more than one pronunciation because the mappings between characters and their pronunciations, in terms of CPs, are not one-to-one, which is also the case with SPs. Pronunciation algorithms are needed to resolve this ambiguity.

## Pronunciation algorithms

In contrast to Arabic, languages such as English and French, with great complexity of mappings from orthography to phonemic sequences may preclude pronunciation algorithms, requiring instead a listing of words with their pronunciations (lexicon).

Our objective is to use Arabic grammatical intra-word and contextual rules to transform one-to-many character-phoneme mapping of Table 2 to one-to-one orthography-pronunciation mapping. We construct a CP pronunciation algorithm that transforms words in MO into CP sequences, which results in a pronunciation dictionary that generally has one-to-one mappings between words in MO and their pronunciations as CP sequences.

We also develop a SP pronunciation algorithm to transform a word in CO into SP sequences, to produce a pronunciation dictionary that has a one-to-one mapping of words in CO with their pronunciations as SP sequences. The alignment method resolves any exceptions to single pronunciations during the training phase of ASR.

As the training set, and hence the language model and the pronunciation dictionary of ASR have MO orthography, and CO is an intermediate form, other processing steps (explained in section “Data preparation and HMM training”) are needed in conjunction with the SP pronunciation algorithm to generate mapping of MO words into SP with one-to-many relationship. A language model and pronunciation dictionary in CO is infeasible because it requires diacritization of text corpus for computation of the LM and generation of a pronunciation dictionary utilizing diacritized words.

The pronunciation algorithms for UG and DG involve parsing of a word written in MO and CO into its respective constituent Graphemes. These representations are used to produce one-to-one dictionaries that list words in MO and their pronunciations in terms of UG pronunciation units, and words in CO and their pronunciations in terms of DG pronunciation units.

The pronunciation rules for Arabic are complex, and hence the CP and SP pronunciation algorithms are described as declarative descriptions to simplify the presentation because the procedural counterparts are complicated by issues related to overlap and precedence among the different rules.

The SP algorithm produces a single pronunciation of a word in CO in all cases. It uses contextual rules to choose a single alternative from among the generic character-phoneme mappings of Table 2. Disambiguation of singleton and geminate phoneme pronunciation of a character depends on the diacritics following the character. Special consideration is made for ‘|’ (آ), and the Tanween characters ‘F’, ‘N’, and ‘K’ () (as each of them is pronounced as a sequence of two SPs; the character sequence ‘wA’ (وا) at the end of a word is mapped into a single SP; each of the characters ‘w’, ‘y’, ‘p’, ‘l’ (ل ة ي و) is mapped into one of the two SP choices of Table 2, depending on context; the character ‘A’ (ا) is mapped into one of three options of Table 2, based on context. The details are provided in section “Simple phoneme pronunciation algorithm”.

The CP algorithm uses intra-word context to reduce the options in Table 2 and is able to produce a single pronunciation of a word in MO in most cases, based on the pronunciation algorithm. The difficulties arise from the Tanween characters and ‘w’, ‘y’, ‘p’ (ة ي و), compounded by lack of diacritics. Additional handling is required for the multiple pronunciations of ‘A’, ‘Al’ and ‘ll’ (لل ال ا), based on Qamariya (coronal) and Shamsiya (non-coronal) context and Hamzat Wasl rules. The explanations are provided in section “Composite phoneme pronunciation algorithm”.

### Simple phoneme pronunciation algorithm

It is useful to provide a literature review of existing methods for grapheme to phoneme mappings before presenting our algorithm. Traditional Arabic literature provides a rich set of pronunciation rules for classical Arabic (Nassir 1985). Recent publications provide pronunciation rules for modern Arabic extracted from traditional sources. However, some of the rules that should be included are excluded, while others that should be left out are incorporated. These have significant consequences on phonetization and recognition accuracy, as these situations arise quite frequently, and hence pronunciation produced using these rules would have many errors.

In recent publications, gemination is handled by doubling a singleton consonant or mapping into its singleton version, which is phonetically inaccurate as demonstrated by geminated plosives which have a single voice onset time and release. Similarly, a long vowel is dealt by doubling its short version, whereas the spectral characteristics of a long vowel are noticeably different from the short vowel counterpart. Also, rules related to Wasl characters ‘w’, ‘k’, ‘f’, ‘b’ (ب ف ك و) are ignored, even though they occur frequently.

Furthermore, four additional forms of ‘ ‘ ‘(ء) which are ‘<‘, ‘>‘, ‘}’, ‘&’ (ؤ ئ أ إ); in addition to ‘|’ (آ); and end of sentence vowels are not addressed (Alghamdi et al. 2004). Mapping of ‘A’ (ا)in the beginning of a sentence into a glottal stop /-/, as well as pronunciation of ‘p’ as/h/in the end sentence are absent from consideration. Handling of short vowels at the end of a sentence by removing them, as well as the case of ‘|’ (آ) is ignored (El-Imam 2004). Situations that require mapping of ‘AF’ (اً) at end of a sentence are not incorporated (Biadsy et al. 2009).

The algorithm presented below builds declarative pronunciation rules to map a word in CO to SP, and includes rules missing in recent literature as mentioned above. We avoid inclusion of abandoned rules in MSA, such as the classical Iqlab rule, as it would result in wrong phonetization and consequently higher word error rates (WER) in speech recognition.

In our algorithm, we make heavy use of diacritics to determine the contextual pronunciation. Thus pronunciation rules regarding ‘A’, ‘Al’, ‘ll’ (ا الـ للـ) at the beginning of a word and ‘p’ (ة) at the end of a word, are covered by diacritics rather than by Shamsiya, Qamariya, and Hamzat Wasl rules (explained in section “Composite phoneme pronunciation algorithm”). These rules are redundant in the presence of precise diacritics found in CO. We assume that ‘G’ (ـ’ـ) has been added to a closed set of words numbering around ten in the pre-processing stage, in which diacritics have been validated. The declarative rules for mapping sequence of character in CO into sequence of SP’s are as follows:

- ‘aAF’ | ‘AF’ -> /a0/; ‘N’ -> /u0/; ‘K’ -> /i 0/
- ‘|’ -> /-a:/
- ‘wAϕ’ -> /u:/
- Identify the mutually exclusive character clusters ـ ‘?’, ‘?o’, ‘?~’, ‘?~o’, ‘?a’, ‘?u’, ‘?i’, ‘?~a’, ‘?~u’, ‘?~i’, ‘?G’, ‘?aG’, ‘?Y’, ‘?aY’, ‘?~G’, ‘?~aG’, ‘?~Y’, ‘?~aY’, where ‘?’ denotes any character except ‘o’, ‘~’, ‘a’, ‘u’, ‘i’, ‘G’, ‘Y’, ‘F’, ‘N’, ‘K’. These clusters identify the intra-word context and yield single pronunciations.
- Map the character clusters according to Table 4. Note that character clusters starting with ‘w’, ‘y’, ‘p’, ‘l’, ‘A’ require special attention. Clusters starting with other characters are treated in the same manner as the character cluster starting with ‘b’.

**Table 4:** Simple phoneme mapping

	**First character of a word**					
**Cluster**	**b**	**w**	**y**	**p**	**l**	**A**
?	"	u:	i:	L	ϕ	Note 1
?A	N/A	u:	N/A	N/A	N/A	N/A
?o	"	f	U	L	W	N/A
?~ or ?~o	**"**	**f**	**U**	**#**	**W**	N/A
?a	" a	f a	U a	# a	W a	N/A
?u	" u	f u	U u	# u	W u	N/A
?i	" i	f i	U i	# i	W i	N/A
?~a	**"** a	**f** a	**U** a	**#** a	**W** a	N/A
?~u	**"**u	**f** u	**U** u	**#** u	**W**u	N/A
?~i	**"**i	**f** i	**U** i	**#** i	**W** i	N/A
?G or ?aG or ?Y or ?aY	" a:	f a:	U a:	# a:	W a:	N/A
?~G or ?~aG or ?~Y or ?~aY	**"** a:	**f** a:	**U** a:	**#** a:	**W** a:	N/A

{-} at sentence beginning; ϕ at word beginning possibly preceded by Wasl characters or their combinations as prefixes; a: otherwise

### Composite phoneme pronunciation algorithm

The declarative rules for mapping a word in MO to CP pronunciation(s) are described below. Pronunciation rules related Shamsiya and Qamariya characters, as well Hamzat Wasl are explicitly incorporated because of the absence of diacritics. Qamariya characters (roughly corresponding to Coronal sounds made using the tip or blade of the tongue) are: ‘ ‘ ‘, ‘>‘, ‘&’, ‘<‘, ‘}’, ‘|’, ‘b’, ‘j’, ‘H’, ‘x’, ‘E’, ‘g’, ‘f’, ‘q’, ‘k’, ‘m’, ‘h’, ‘w’, ‘y’; the Shamsiya characters are: ‘t’, ‘v’, ‘l’, ‘d’, ‘r’, ‘$’, ‘s’, ‘Z’, ‘S’, ‘D’, ‘T’, ‘Z’, ‘n’. Hamzat Wasl rules govern the pronunciation of ‘A’.

- Add the character ‘G’ at the appropriate positions in the known set of words.
- ‘G’ | ‘Y’ ->{a:}
- ‘|’ ->{-}{a:}
- ‘ ‘ ‘ | ‘>‘ | ‘&’ | ‘<‘ | ‘}’ -> {-}
- ‘n’ | ‘F’ | ‘qA’ | ‘N’ | ‘K’ -> {0}. Note that ‘F’ is pronounced as /a 0/, but we do not need to define a distinct CP as /a 0/ because the short vowel is part of the CP of the previous character(s), and the characters ‘F’, ‘N’, ‘K’ occur only at the end of a word. Similar observations hold for the characters ‘N’ and ‘K’
- ‘p’ in ‘pF’ | ‘pN’ | ‘pK’ ->{#}
- ‘wAϕ’ ->{u:} | {f}{a:}
- ‘w’ ->{f} in the following character sequences: ‘ϕw’, ‘wF’, ‘wN’, ‘wK’, ‘w|’, ‘|w’, ‘wG’, ‘Gw’, ‘wY’, ‘wp’, ‘wAl’, ‘w<‘, ‘<w’
- ‘w’ ->{f} in the following character sequences at the beginning of a word: ‘Alw’
- ‘w’ ->{u:} in the following character sequences: ‘&w’
- ‘y’ ->{U} in the following character sequences: ‘ϕy’, ‘yF’, ‘yN’, ‘yK’, ‘y|’, ‘|y’, ‘yG’, ‘Gy’, ‘yY’, ‘yp’, ‘yAl’
- ‘y’ ->{U} in the following character sequences at the beginning of a word: ‘Aly’
- ‘y’ ->{i:} in the following character sequences: ‘y&’, ‘&y’, ‘y<‘, ‘<y’, ‘y}’, ‘}y’, ‘yA’
- ‘ll’ ->{W} in the following character sequences at the beginning of a word: ‘ll’ ‘wll’, ‘fll’, ‘kll’, ‘fkll’, or ‘wkll’, provided the character sequence is followed by a Shamsiyah sound
- ‘Al’ -> {ϕ} in the following character sequences at the beginning of a word: ‘wAl’, ‘bAl’, ‘fAl’, ‘wbAl’, ‘fbAl’, ‘kAl’, ‘fkAl’, or ‘wkAl’, provided the character sequence is followed by a Shamsiyah sound
- ‘Al’ ->{-} in the following character sequences at the beginning of a sentence: ‘Al’, provided the character sequence is followed by a Shamsiyah sound
- ‘Al’ ->{-}{W} in the following character sequences at the beginning of a sentence: ‘Al’, provided the character sequence is followed by a Qamariyah sound
- ‘A’ ->{-} at the beginning of a sentence
- ‘A’ ->{a:} in the middle of a word
- ‘A’ ->{a:} | {ϕ} at the end of a word
- ‘A’ ->{ϕ} at the beginning of a word possibly preceded by Wasl characters
- Map the remaining characters according to Table 2.

### Illustrative application of algorithms

We explain below transformation of the word ‘waAsotamaEuwA’ (وَاسْتَمَعُوا) in CO which is ‘wAstmEwA’ (واستمعوا) in MO.

The word ‘waAsotamaEuwA’ in CO is transformed into the SP sequence / fa@#a.aMu: /as follows: Since ‘w’ is followed by ‘a’, the cluster ‘a’ is pronounced as /wa/ rather than ‘w’ being pronounced as /u:/. ‘A’ is pronounced as /ϕ/ because it is preceded by Wasl character ‘w’ discounting its transformation into /-/ or /a:/ if no contextual rules are used. Regular characters ‘s’, ‘t’, ‘m’, ‘E’ and hence their clusters with the succeeding diacritics are pronounced according to the second column of Table 4. The cluster ‘wAϕ’ is pronounced as /u:/ according to the rules presented in the first subsection on SP pronunciation algorithm, but would have been wrongly pronounced as /u: a:/ or /f a:/ or /u: ϕ/ or /f ϕ/, or /u: -/ or /f -/ without intra-word contextual rules. The large number of pronunciation options in the absence of contextual rules may not be disambiguated correctly during the alignment phase.

The word ‘wAstmEwA’ in MO is transformed into two CP sequence{ f@#.Mu: }and{ f@#.Mwa: } as follows: ‘w’ is mapped to {w}; ‘A’ is pronounced as /ϕ/ because it is preceded by Wasl character’w’, rather than {a:} or {-} if no contextual rules are used; the regular characters ‘s’, ‘t’, ‘m’, and ‘E’are mapped into {w}, {@}, {#}, {.}, and {M} respectively; the characters ‘wA’ are mapped to {w} and {a:} individually and { u:} together. The correct option of pronunciation is determined during the alignment phase of training.

## Data preparation and HMM training

HMM training and recognition performance evaluation for the variety of ASRs (Uniform and Bigram Language Model and Context Independent and Dependent with SP (SP_A and SP_M), CP, DG, and UG) require preparation of data from a speech corpus transcribed in CO in order to skip the diacritization stage required for SP_M pronunciation for a corpus in MO, and to avoid confounding of the performance of the phonetic unit with diacritization quality of MO to CO. Training and recognition for the SP_A pronunciation unit is conducted after diacritizing the MO version of the corpus with the MADA toolkit (Habash and Roth 2009).

We choose two Arabic speech corpora for empirical experiments, as these specifically have Modern Standard Arabic and all the text is in Classical Orthography: A-SpeechDB and SAAVB—both having prompted utterances in MSA with CO label files manually reviewed according to actual pronunciation. As the experimental results for both corpora lead to consistent conclusions, we present only results related to A-SpeechDB. The dissimilarity is that corresponding values of WER for SAAVB is higher than A-SpeechDB by 10–15 points in absolute terms because the bandwidth of SAAVB telephone recording is 3.5 kHz compared to 8 kHz microphone recording for A-SpeechDB (Alghamdi et al. 2005; ELRA 2011).

A-SpeechDB consists of 24,000 MO unique words (35,000 CO words) spoken by 122 male speakers, recorded using a microphone at 16 kHz sampling rate, 16 bit PCM. The SAAVB corpus consists of 1719 MO unique words (3125 CO words) spoken by 484 male speakers, spoken over cellular telephones, received by land telephones in a quiet environment and sampled at 8 kHz, 16 bit PCM.

Data preparation is based on word label files and word listings in both MO and CO. These are used to create pronunciation label files in terms of SP_M, SP_A, CP, DG, and UG for use in training, and construct pronunciation dictionaries for use in the recognition experiments. Note that the contextual pronunciation algorithm is used at the training phase and the pronunciation dictionary at the recognition phase, in contrast to English which uses pronunciation dictionary at both the training and recognition stages.

The pronunciation label files are constructed for SP_M and DG from the word label files in CO and the pronunciation files for SP_A, CP and UG are built from the MO word label files. The SP (for SP_A and SP_M) and CP contextual pronunciation algorithms are utilized to obtain the pronunciation files.

The pronunciation dictionaries constructed are: MO:SP_A, MO:SP_M, MO:CP, MO:UG, and MO:DG. The MO:CP pronunciation dictionary is obtained by applying the CP pronunciation algorithm to the MO transcription. The MO:SP_A pronunciation dictionary is built by the application of the SP pronunciation algorithm to the corresponding multiple words in CO. The pronunciation probabilities for a word in the MO:SP_A dictionary are provided by the statistics of occurrence of the CO words corresponding to the word in MO. The UG and DG dictionaries are constructed by the parsing of CO and MO transcriptions.

We train HMMs for both the context independent (CI) and context dependent (CD) ASR. The acoustic units trained for context independent are SP_A, SP_M, SP_M1, CP, UG, and DG. SP_M1 is the manually diacritized simple phoneme with single emitting state HMM for short vowels. For the context dependent ASR systems, we implement both data-driven and decision tree approaches for clustering of the context dependent pronunciation units. The data-driven method has the advantage that it is readily applicable to both phonetic and grapheme pronunciation units and can be uniformly implemented across SP_A, SP_M, SP_M1, CP, DG, and UG. This method has the disadvantage that it is not able to extrapolate to units outside the training set. Decision trees need to be constructed for each of the pronunciation units, and their application to grapheme methods requires extrapolation of graphemes to phonetic classifications. The decision tree method has the advantage of allowing unseen units to be clustered.

In the data-driven approach, we use a threshold of 100 as the minimum occupancy and greatest distance between any two states in cluster of 350 to cluster 4000 word-internal units to approximately 750 tied units. The threshold value ensures sufficient data for statistically valid experiments and the values are comparable to other studies (Young et al. 1994). For the decision tree approach, we set the increase in likelihood threshold parameter of HTK to 350 in order to yield approximately 750 tied units, making it comparable to the data-driven approach.

The SP_M, SP_A and SP_M1 decision trees utilize phonetic classifications similar to the 200 queries of HTK RM demo (Young et al. 2006). Adjustments are made for the specific phonemes of Arabic, including uvular, pharyngeal, and glottal sounds, and singleton and geminated versions of a sound are grouped. The CP decision tree is built from the SP_A, SP_M, and SP_M1 decision tree by classifying a consonant short vowel cluster according to the consonant, and omitting geminated sounds and short vowels.

The UG decision tree extrapolates graphemes to phonetic classifications using generic mappings of Table 2. Graphemes with simple pronunciations are classified according to their phonetic realization. For example, ‘s’ and ‘z’ (ز س) are grouped together because the pronunciations of these Graphemes are fricative, central, coronal, anterior, continuant, and strident. Complex pronunciations (i.e., multiple or sequence pronunciation) of the graphemes ‘F’, ‘N’, ‘K’, A’, ‘|’, ‘p’, ‘l’, ‘w’, ‘y’ () ideally require developing multiple versions of decision trees for various classification options, and then choosing the best according to recognition performance. Because we need a single decision tree for UG, the following choices are made according to the most recurring pronunciation in our corpus: ‘w’ and ‘y’ (vowels rather than glides), ‘l’ (glide rather thanϕ), ‘p’ (dental stop rather than glottal fricative), ‘|’ (glottal stop rather than vowel), ‘A’ (long vowel rather than glottal stop or ϕ), ‘F’, ‘N’, and ‘K’ (nasal rather than vowels). The DG decision tree is developed in a similar manner, with a grapheme followed by ‘~’ treated as a single geminated grapheme.

In order to implement statistically valid training and recognition tasks, we use the K-fold method with three folds to partition the data, while taking speaker independence into account (Blum et al. 1999).

Bigram is estimated from the transcriptions in MO for the recognition set of an experiment, rather than the entire set to avoid any bias. Results are obtained by averaging the recognition performance values for the three folds.

In keeping with standard practice, training is conducted using the flat-start incremental methodology of the HTK toolkit (Young et al. 2006). We use left-to-right non-skip HMM with continuous Gaussian density Mel Frequency Cepstral Coefficients (MFCC) and a diagonal covariance matrix. The waveforms are pre-emphasized using a first order FIR filter with a coefficient of 0.97, and windowed using Hamming windows of size 25 ms that are 10 ms apart. The frames are transformed into sequences of twelve MFCC, supplemented with an energy coefficient and their first- and second-order derivatives, to produce feature vectors with a length of thirty-nine.

The standard SP HMM has three emitting states. As the CP contains consonant short vowel clusters, the corresponding HMM potentially requires more states to model the additional acoustic events for the short vowel. Since the absorbed vowel is short and has fewer acoustic events than the consonant, we expect the CP HMM to have four or five emitting states, with one or two of the emitting states corresponding to the short vowel, and the remaining for the consonant. Four emitting states are more likely than five because of the shortness of the vowel. Naturally, the enlarged number of states can accommodate CPs that are equivalent to SPs, as in the case of the long vowels /a:/,/i:/,/u:/. Based on the argument above, SP_M1 is also investigated to examine the effect of modeling short vowels with single emitting states on SP (Soltau et al. 2009).

Our experiments train CI and CD HMMs with 3-5 emitting states (and 1 emitting state for short vowels in SP_M1), and 1–12 mixtures. Examination of the number of frames allocated to a state in an HMM with a single mixture indicates that we have enough data to produce statistically valid estimates. Furthermore, the various fold experiments yield similar results. Default HTK settings are used for purposes of training and recognition of all the ASRs, and no fine tuning of any specific ASR is applied.

## Recognition and performance

We conduct empirical experiments to study recognition performance of the various ASR systems for a number of emitting states and mixtures. As our objective is to compare the recognition performance of SP_A, SP_M, SP_M1, CP, UG, and DG, we do not conduct fine tuning or optimization for each pronunciation unit individually for reasons explained in the "Background".

The recognition process utilizes the token-passing Viterbi search algorithm and dynamic programming to compute WER. We compute the average WER for each of the ASRs’ three folds and note that the variations in the performance between the folds are insignificant. The number of (emitting) states of CI HMMs that yield the best performance over the range of 1–12 mixtures for Uniform LM are as follows: CP: five; SP_A, SP_M, SP_M1: four; UG: five; and DG: four. The number of states that yield the best performance with Bigram are as follows: CP: four; SP_A, SP_M, SP_M1: three; UG: four; and DG: three. For the CD HMMs, the optimal number of states is the same as their CI counterparts.

The recognition results show that the HMM for the CP has an extra state compared to that of the SP, suggesting that the short vowel component of the consonant centric cluster requires a single state to model. In our experiments, recognition performances of CD ASRs with decision tree clustering are comparable to those with data-driven clustering, and hence are not plotted in the figures. This is in agreement with published studies on Triphones (Young et al. 1994; Beulen et al. 1997).

**Fig. 1:**
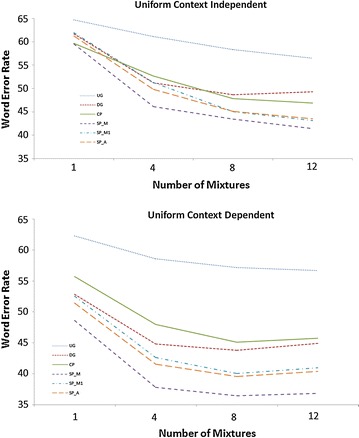
WER of ASR system with Uniform LM. *Top plot*: Context Independent; *Bottom plot*: Context Dependent. Undiac Grapheme (UG) 5 emitting states (*dot*); Diac Grapheme (DG) 4 emitting states (*short dash*); Composite Phoneme (CP) 5 emitting states (*solid*); Simple Phoneme Manual Diacritization (SP_M) 4 emitting states (*medium dash*); Simple Phoneme Manual Diacritization with single state short vowels (SP_M1) 4 emitting states (*medium dash dot*); Simple Phoneme Automatic Diacritization (SP_A) 4 emitting states (*long dash*)

**Fig. 2:**
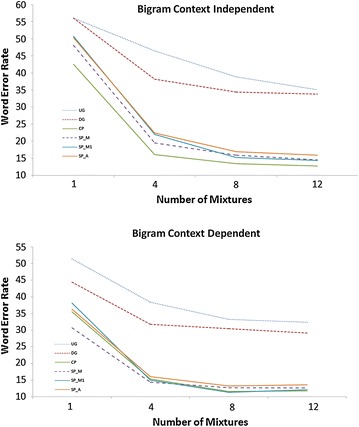
WER of ASR system with Bigram LM. Top plot: Context Independent; *Bottom plot*: Context Dependent. Undiac Grapheme (UG) 4 emitting states (*dot*); Diac Grapheme (DG) 3 emitting states (*short dash*); Composite Phoneme (CP) 4 emitting states (*solid*); Simple Phoneme Manual Diacritization (SP_M) 3 emitting states (*medium dash*); Simple Phoneme Manual Diacritization with single state short vowels (SP_M1) 3 emitting states (*medium dash dot*); Simple Phoneme Automatic Diacritization (SP_A) 3 emitting states (*long dash*)

Figures 1 and 2 graph WER of ASRs versus mixtures for the optimal number of HMM states. The left plot is for CI pronunciation units and the right plot is for CD pronunciation units. In all cases the word error rate calculation is based on the undiacritized forms (NIST style scoring) (Saon et al. 2010).

Figure 1 graphs the WER for ASRs with Uniform LM for CI (left) and CD (right). The CP with five states has a minimum WER of 46.89 % for CI and 45.13 % for CD. The SP_M, SP_A, and SP_M1 with four states have minimum WERs of 41.38, 43.48, and 43.09 % respectively for CI and 36.44, 39.55, and 40.05 % for CD. The UG with five states has a minimum WER of 56.48 % for CI and 56.72 % for CD; and DG with four states has a minimum WER of 48.65 % for CI and 43.80 % for CD.

Figure 2 graphs the WER for ASR with the Bigram LM for CI (left) and CD (right). The CP with four states has a minimum WER of 12.70 % for CI and 11.64 % for CD. The SP_M, SP_A, and SP_M1 with three states have minimum WERs of 14.58, 15.94, and 14.39 % respectively for CI and 12.64, 13.22 %, and 11.31 % for CD. The UG with four states has a minimum WER of 35.06 % for CI and 32.40 % for CD. The DG with three states has a minimum WER of 33.78 % for CI and 29.17 % for CD.

The recognition performance results suggest that Phonetic methods (CP, SP) are better than Grapheme methods (DG, UG) typically. For Uniform LM: CP performs worse than all SP’s (SP_M, SP_A, SP_M1) and SP_M has best performance for both CI and CD.

For Bigram LM: CP has best performance for CI and CP has best performance along with SP_M1 for CD. The automated diacritized SP_A has higher WER than the manual diacritized SP_M and SP_M1 for both CI and CD. For practical use, however, CP is better than SP_M1 and also SP_M and SP_A because it does not require diacritization.

The scalability of the CP for larger vocabulary is tested by conducting recognition on a vocabulary of 24,000 MO words with four emitting states for CP and three emitting states for SP. The WER’s are 13.69, 15.08, and 16.86 % for CP, SP_M, and SP_A respectively

The Phonetic methods outperform their Grapheme counterparts because they explicitly cater to the complexities of character-phoneme mappings shown in Table 2 by using pronunciation algorithms that yield single pronunciations of words. The Grapheme methods assume a one-to-one character-phoneme mapping, treating Arabic as a phonemic language (i.e., a language with a shallow orthography), and rely on training and context dependent units to implicitly compensate for that erroneous assumption. Even though the Grapheme methods gain more from the context dependent units, they are unable to match the performance of the Phonetic methods.

More specifically, the degradation in recognition performance in the Grapheme methods results from the following frequently-occurring discrepancies between the actual relationships between orthography and pronunciation and those represented by the Grapheme methods. The Phonetic methods incorporate language-specific knowledge, which results in better performance.

1. The characters ‘>‘, ‘&’,’<‘, ‘}’ (ئ إ ؤ أ) have the same pronunciation as ‘ ‘ ‘ (ء) and hence are mapped onto the same HMM in SP and CP methods. The Grapheme methods define multiple HMMs for the graphemes and thus have higher variability.
2. Each of the characters ‘w’, ‘y’, ‘A’, ‘l’ (ل ا ي و) has more than one pronunciation, including an option of no pronunciation for ‘A’ and ‘l’ (ل ا). Each pronunciation is mapped onto a distinct HMM in Phonetic methods, whereas they are lumped into a single HMM in the Grapheme methods and therefore result in HMMs that are less coherent.
3. The character ‘|’ (آ) is pronounced as a sequence of a consonant (glottal stop) and a long vowel. Each member of the sequence has its own HMM in Phonetic methods, whereas the Grapheme methods utilize a single new HMM for the sound sequence.
4. The character ‘p’ (ة) has two pronunciations, which can be defined in terms of other HMMs in the Phonetic methods. The Grapheme methods define a new HMM for the grapheme with a single mapping, thereby resulting in the same problems as in points 1 and 2.
5. The characters ‘F’, ‘N’, and ‘K’ (ــٍ ــٌ ــً) are treated as sequences of a short vowel and consonant for SP and as a single pronunciation for CP. The Grapheme methods do not treat these as special cases and define distinct new HMMs for each of these graphemes.

The above differences between Phonetic and Grapheme pronunciation units manifest themselves in better performance for Phonetic methods. We have observed that in several cases UG inaccurately recognizes words, replacing ‘|’ (آ) with ‘A’ (ا) and ‘p’ (ة) with ‘v’(ث), and leaves out the end-of-word Tanween characters ‘F’, ‘N’, and ‘K’ (ــٍ ــٌ ــً). Replacement of ‘|’ (آ) with ‘A’ (ا) is most likely because آ (‘|’) is represented with a single HMM that is dominated with the long vowel, causing the confusion; ‘F’, ‘N’ and ‘K’ (ــٍ ــٌ ــً) are left out in the recognized words because the closest words are those without these Tanween characters; the error in recognizing ‘p’ (ة) as ‘v’ (ث) is probably the result of HMM modeling a combination of /#/ and /N/ as a sound close to />/.

The multitude of departures of the SP and CP methods from the DG and UG methods manifest the fundamental differences between the Phonetic and Grapheme methods, demonstrating that SP is not simply a special case of DG nor CP a special case of UG, with a few added rules. Not only are the complex pronunciation algorithms of the Phonetic methods different from the simple 1-1 pronunciation mappings of Grapheme methods, but the empirical recognition performance and the segmentation properties of pronunciation units are also different.

## Conclusion

This paper proposes the concept of Composite Phoneme (CP), as opposed to Simple Phonemes (SP) which are used in existing phoneme-based ASR systems. In this paper, Arabic intra-(“word contextual grammatical rules are used to generate SP pronunciations from diacritized words, and to generate CP pronunciations from undiacritized words—with most words having single pronunciations. Empirical experiments show that CPs is a promising pronunciation unit for Arabic with lower error rates.
